# Does parity matter in women’s risk of dementia? A COSMIC collaboration cohort study

**DOI:** 10.1186/s12916-020-01671-1

**Published:** 2020-08-05

**Authors:** Jong Bin Bae, Darren M. Lipnicki, Ji Won Han, Perminder S. Sachdev, Tae Hui Kim, Kyung Phil Kwak, Bong Jo Kim, Shin Gyeom Kim, Jeong Lan Kim, Seok Woo Moon, Joon Hyuk Park, Seung-Ho Ryu, Jong Chul Youn, Dong Young Lee, Dong Woo Lee, Seok Bum Lee, Jung Jae Lee, Jin Hyeong Jhoo, Juan J. Llibre-Rodriguez, Jorge J. Llibre-Guerra, Adolfo J. Valhuerdi-Cepero, Karen Ritchie, Marie-Laure Ancelin, Isabelle Carriere, Ingmar Skoog, Jenna Najar, Therese Rydberg Sterner, Nikolaos Scarmeas, Mary Yannakoulia, Efthimios Dardiotis, Kenichi Meguro, Mari Kasai, Kei Nakamura, Steffi Riedel-Heller, Susanne Roehr, Alexander Pabst, Martin van Boxtel, Sebastian Köhler, Ding Ding, Qianhua Zhao, Xiaoniu Liang, Marcia Scazufca, Antonio Lobo, Concepción De-la-Cámara, Elena Lobo, Ki Woong Kim, for Cohort Studies of Memory in an International Consortium (COSMIC), Perminder S. Sachdev, Darren M. Lipnicki, Steve R. Makkar, John D. Crawford, Anbupalam Thalamuthu, Nicole A. Kochan, Yvonne Leung, Jessica W. Lo, Yuda Turana, Erico Castro-Costa, Bagher Larijani, Iraj Nabipour, Kenneth Rockwood, Xiao Shifu, Richard B. Lipton, Mindy J. Katz, Pierre-Marie Preux, Maëlenn Guerchet, Linda Lam, Ingmar Skoog, Toshiharu Ninimiya, Richard Walker, Hugh Hendrie, Antonio Guaita, Liang-Kung Chen, Suzana Shahar, Jacqueline Dominguez, Murali Krishna, Mary Ganguli, Kaarin J. Anstey, Michael Crowe, Mary N. Haan, Shuzo Kumagai, Tze Pin Ng, Henry Brodaty, Kenichi Meguro, Richard Mayeux, Nicole Schupf, Perminder Sachdev, Mary Ganguli, Ronald Petersen, Richard Lipton, Edwin S. Lowe, Karen Ritchie, Ki-Woong Kim, Louisa Jorm, Henry Brodaty

**Affiliations:** 1 Department of Neuropsychiatry, Seoul National University Bundang Hospital, Seongnam, South Korea; 2 Department of Psychiatry, College of Medicine, Seoul National University, Seoul, South Korea; 3 Centre for Healthy Brain Ageing, University of New South Wales, Sydney, Australia; 4 Dementia Collaborative Research Centre, University of New South Wales, Sydney, Australia; 5 Department of Psychiatry, Yonsei University Wonju Severance Christian Hospital, Wonju, South Korea; 6 Department of Psychiatry, Dongguk University Gyeongju Hospital, Gyeongju, South Korea; 7 Department of Psychiatry, School of Medicine, Gyeongsang National University, Jinju, South Korea; 8 Department of Neuropsychiatry, Soonchunhyang University Bucheon Hospital, Bucheon, South Korea; 9 Department of Psychiatry, School of Medicine, Chungnam National University, Daejeon, South Korea; 10 Department of Psychiatry, School of Medicine, Konkuk University and Konkuk University Chungju Hospital, Chungju, South Korea; 11 Department of Neuropsychiatry, Jeju National University Hospital, Jeju, South Korea; 12 Department of Psychiatry, School of Medicine, Konkuk University and Konkuk University Medical Center, Seoul, South Korea; 13 Department of Neuropsychiatry, Kyunggi Provincial Hospital for the Elderly, Yongin, South Korea; 14 Department of Neuropsychiatry, Seoul National University Hospital, Seoul, South Korea; 15 Department of Neuropsychiatry, Inje University Sanggye Paik Hospital, Seoul, South Korea; 16 Department of Psychiatry, Dankook University Hospital, Cheonan, South Korea; 17 Department of Neuropsychiatry, Kangwon National University Hospital, Chuncheon, South Korea; 18 Finlay-Albarrán Faculty of Medical Sciences, Medical University of Havana, Havana, Cuba; 19 Institute of Neurology and Neurosurgery, Havana, Cuba; 20 Medical University of Matanzas, Matanzas, Cuba; 21 Memory and Aging Center, University of California San Francisco, San Francisco, CA USA; 22 Inserm U1061 Neuropsychiatry: Epidemiological and Clinical Research, La Colombière Hospital, Montpellier, France; 23 Department of Clinical Brain Sciences, University of Edinburgh, Edinburgh, UK; 24 Department of Psychiatry and Neurochemistry, Institute of Neuroscience of Physiology, Sahlgrenska Academy, Centre for Ageing and Health (AGECAP) at the University of Gothenburg, Gothenburg, Sweden; 25 1st Department of Neurology, Aiginition Hospital, National and Kapodistrian University of Athens, Medical School, Athens, Greece; 26 Department of Neurology, Columbia University, New York, USA; 27 Department of Nutrition and Dietetics, Harokopio University, Athens, Greece; 28 Neurology Department, University Hospital of Larissa, University of Thessaly, Larissa, Greece; 29 Geriatric Behavioral Neurology, Tohoku University, Sendai, Japan; 30 Institute of Social Medicine, Occupational Health and Public Health (ISAP), Medical Faculty, University of Leipzig, Leipzig, Germany; 31 Department of Psychiatry and Neuropsychology, School for Mental Health and Neuroscience (MHeNs), Maastricht University, Maastricht, Netherlands; 32 Institute of Neurology, Huashan Hospital, Fudan University, Shanghai, China; 33 National Clinical Research Center for Aging and Medicine, Huashan Hospital, Fudan University, Shanghai, China; 34 University of São Paulo, São Paulo, Brazil; 35 Universidad de Zaragoza, Zaragoza, Spain; 36 Instituto de Investigación Sanitaria Aragón, Zaragoza, Spain; 37 Biomedical Research Networking Center for Mental Health Network (CIBERSAM), Instituto de Salud Carlos III, Madrid, Spain; 38 Department of Brain and Cognitive Science, Seoul National University College of Natural Sciences, Seoul, South Korea

**Keywords:** Dementia, Alzheimer’s disease, Risk factors, Parity, Women

## Abstract

**Background:**

Dementia shows sex difference in its epidemiology. Childbirth, a distinctive experience of women, is associated with the risk for various diseases. However, its association with the risk of dementia in women has rarely been studied.

**Methods:**

We harmonized and pooled baseline data from 11 population-based cohorts from 11 countries over 3 continents, including 14,792 women aged 60 years or older. We investigated the association between parity and the risk of dementia using logistic regression models that adjusted for age, educational level, hypertension, diabetes mellitus, and cohort, with additional analyses by region and dementia subtype.

**Results:**

Across all cohorts, grand multiparous (5 or more childbirths) women had a 47% greater risk of dementia than primiparous (1 childbirth) women (odds ratio [OR] = 1.47, 95% confidence interval [CI] = 1.10–1.94), while nulliparous (no childbirth) women and women with 2 to 4 childbirths showed a comparable dementia risk to primiparous women. However, there were differences associated with region and dementia subtype. Compared to women with 1 to 4 childbirths, grand multiparous women showed a higher risk of dementia in Europe (OR = 2.99, 95% CI = 1.38–6.47) and Latin America (OR = 1.49, 95% CI = 1.04–2.12), while nulliparous women showed a higher dementia risk in Asia (OR = 2.15, 95% CI = 1.33–3.47). Grand multiparity was associated with 6.9-fold higher risk of vascular dementia in Europe (OR = 6.86, 95% CI = 1.81–26.08), whereas nulliparity was associated with a higher risk of Alzheimer disease (OR = 1.91, 95% CI 1.07–3.39) and non-Alzheimer non-vascular dementia (OR = 3.47, 95% CI = 1.44–8.35) in Asia.

**Conclusion:**

Parity is associated with women’s risk of dementia, though this is not uniform across regions and dementia subtypes.

## Background

Dementia is one of many disorders with sex and gender differences in epidemiology, risk factors, and outcomes. Compared to men, women show a greater prevalence of dementia [1], a higher risk of Alzheimer disease (AD) conferred by the apolipoprotein ε4 allele [2], and faster loss of autonomy after diagnosis of AD [3]. Pregnancy and childbirth are distinctive experiences of women and are associated with changes in hormone levels, health conditions, and lifestyle that influence the risk of dementia. For example, estrogen is closely associated with the risk of AD and vascular dementia (VaD) [4], and its serum level increases more than 10-fold in the third trimester of pregnancy [4, 5]. A higher number of births is generally associated with lower socioeconomical status and educational level, factors that may increase the risk of dementia [6]. Additionally grand multiparity (5 or more childbirths) increases the chances of developing hypertension, coronary heart disease, and diabetes mellitus (DM) [7–9], all of which can increase the risk of dementia.

Studies on whether parity affects the risk of dementia are limited, and their results are different according to study design and study population with different level of parity. Some research suggests that more childbirths increases the risk of dementia. A pooled analysis of two population-based cohorts from South Korea and Greece found that grand multiparous women had a 1.7-fold higher risk of AD than women with 1 to 4 childbirths [10], and a case-control study in Germany found that nulliparity was less frequent in women with AD than in cognitively normal women [11]. However, a prospective cohort study in Italy found nulliparous women to show a comparable risk of dementia to women with 1 to 4 childbirths [12], and an analysis of a claims database from the USA found that women with 3 or more childbirths showed a lower risk of dementia than women giving birth only once (primiparous) [13].

In this study, we used the pooled data of 11 population-based prospective cohort studies from 11 countries over 3 continents to investigate whether parity influences the risk of dementia, and whether there are differences associated with geographic region and type of dementia. Knowing whether parity affects the risk of dementia will help to clarify sex and gender difference in dementia and could identify a high-risk group in women. With such a diverse overall sample, our study might also help to explain the different results from previous studies.

## Methods

### Study population

We pooled baseline data for community-dwelling women aged 60 or older from 11 members of the Cohort Studies of Memory in an International Consortium (COSMIC) collaboration (Table 1) [14–25]. The participants were randomly sampled in all studies but the Maastricht Ageing Study (MAAS). Although the Leipzig Longitudinal Study of the Aged (LEILA 75+) included both community-dwelling and institutionalized participants, we included the community sample only in the present study. The included cohorts varied in size from 342 to 3919 participants. From an initial sample of 16,296 women, we excluded 1504 who did not have data on educational level, hypertension, DM, or parity, giving a final sample of 14,792 women.

**Table 1:** Contributing cohorts

Study	Location	Start	Ethnicity*	Participants^†^ (all/women/included)	Age^‡^	Parity^‡^	Dementia^†^ (all/AD/VaD)
CHAS^15^	Havana and Matanzas, Cuba	2003	Cuban	2944/1913/1552	75.1 ± 7.2 (65–100)	2.5 ± 2.2 (0–23)^c^	183/105/11
ESPRIT^16^	Montpellier, France	1999	White	2259/1313/1094	72.5 ± 5.6 (65–93)	2.4 ± 1.5 (0–9)^cb^	19/17/1
H70^17^	Gothenburg, Sweden	2000	White	1016/787/575	74.7 ± 5.4 (70–92)	2.0 ± 1.6 (0–20)^c^	16/7/4
HELIAD^18^	Larissa and Marousi, Greece	2009	White	1814/1074/918	72.1 ± 5.5 (60–93)	2.0 ± 0.8 (0–7)^cb^	22/16/3
KLOSCAD^19^	Nationwide, South Korea	2010	Asian	6818/3919/3686	70.7 ± 7.2 (60–101)	3.6 ± 1.7 (0–12)^cb^	190/145/17
KS^20^	Kurihara, Japan	2008	Asian	590/365/365	80.3 ± 4.2 (74–94)	2.4 ± 1.3 (0–7)^cb^	49/32/3
LEILA 75+^21^	Leipzig, Germany	1997	White	1265/964/636	81.2 ± 4.9 (75–99)	1.6 ± 1.3 (0–10)^c^	68/48/14
MAAS^22^	South Limburg, Netherlands	1993	White	683/342/342	69.2 ± 6.2 (60–82)	2.6 ± 1.9 (0–9)^c^	26/n.a./n.a.
SAS^23^	Shanghai, China	2010	Asian	3141/1873/1869	71.2 ± 8.4 (60–100)	2.0 ± 1.4 (0–9)^cb^	97/67/7
SPAH^24^	Sao Paulo, Brazil	2003	Brazilian	2072/1255/1149	72.6 ± 6.4 (65–101)	5.6 ± 4.1 (0–24)^c^	68/24/13
ZARADEMP^25^	Zaragoza, Spain	1994	White	4638/2683/2606	74.5 ± 9.6 (60–102)	2.4 ± 1.9 (0–12)^cb^	152/n.a./n.a.

*CHAS* Cuban Health and Alzheimer Study, *ESPRIT* Etude SanteÂ Psychologique et Traitement, *H70* Gothenburg H70 Birth Cohort Studies, *HELIAD* Hellenic Longitudinal Investigation of Aging and Diet, *KLOSCAD* Korean Longitudinal Study on Cognitive Aging and Dementia, *KS* Kurihara Study, *LEILA75+* Leipzig Longitudinal Study of the Aged, *MAAS* Maastricht Ageing Study, *SAS* Shanghai Aging Study, *SPAH* Sao Paulo Ageing & Health Study, *ZARADEMP* Zaragoza Dementia Depression Project, *AD* Alzheimer’s disease, *VaD* vascular dementia, *n.a.* not available

The participants of all studies but MAAS were randomly sampled.

*Ethnicity of the country

^†^Numbers at the baseline assessment

^‡^Mean ± standard deviations (range)

^c^Number of children

^cb^Number of childbirths

### Measures

All studies provided data on dementia diagnosis, based on DSM-IV criteria [26] in 10 studies and DSM-III-R criteria [27] in one. Nine studies provided data on dementia subtypes: AD according to the NINCDS-ADRDA criteria [28] or DSM-IV criteria [26], and VaD according to the NINDS-AIREN criteria [29] or DSM-IV criteria [26].

Other data included parity, age, sex, educational level, and presence of hypertension and DM which were all harmonized when necessary. We assigned parity as the number of childbirths in 6 cohorts and as the number of children in 5 cohorts. When the participants adopted and fostered children or their children died after birth, the number of childbirths and children could be different. However, only 3 among 590 women (0.3%) had different numbers for childbirths and children in Kurihara study (KS) providing numbers for both childbirths and children, and we used data on childbirth in these 3 cases. To harmonize data on educational level, we classified it into 3 categories. For nine studies with data on years of education, we classified educational level as ≤ 6, 7 to 12, or >  12 years. Three comparable education level groups were used for the remaining two studies with categorical data: primary school or under, secondary school, and tertiary school or above. For hypertension and DM, we used all available information from a study relevant to diagnosing or classifying the condition. In a study with limited information, hypertension and DM may be classified only from a medical history record, while for another study, it may be indicated by any of self-reported history, use of relevant medication, or measured blood pressure or glucose level exceeding values indicated by international guidelines. Four studies (Cuban Health and Alzheimer Study [CHAS], Hellenic Longitudinal Investigation of Aging and Diet [HELIAD], Korean Longitudinal Study on Cognitive Aging and Dementia [KLOSCAD] and Sao Paulo Ageing & Health Study [SPAH]) additionally provided data on economic status, employment, and length of reproductive years. Low economic status was defined as lower 50% based on assets in the CHAS, less than KRW 1,000,000 of monthly house income in the KLOSCAD, and BRL 360 or less of monthly house income in the SPAH. In the HELIAD, 4 socioeconomic variables regarding a set of economic reserves or assets, holiday travelling, and leisure time activities were used to define low economic status [30]. Unemployment was defined as “never been employed in lifetime” in all cohorts. The length of reproductive years was calculated by subtracting the age at menarche from the age at menopause.

### Analysis

We initially examined the association between parity (0, 2, 3, 4, ≥ 5 vs 1) and the risk of all-cause dementia with unadjusted and adjusted binary logistic regressions. The adjusted model included age, educational level, hypertension, DM, and cohort as covariates. We employed primiparous women as the reference instead of nulliparous women because nulliparity is associated with earlier menopause [31], and women with earlier menopause had an increased risk of dementia [32]. In obstetrics, 5 or more parities and 10 or more parities are defined as grand multiparity and great grand multiparity, respectively, and they were known to increase obstetric and neonatal complications [33]. Because the number of women with 5 or more births was relatively low, we did not analyze each number of births from 5 or more separately, but rather grouped them into a grand multiparity group. In addition, we further classified grand multiparous women who gave 10 or more childbirths as a great grand multiparity group to examine whether an extremely higher number of parity additionally increase the risk of dementia. Next, we collapsed women with 1 to 4 parities into a referent group and compared this against nulliparity and grand multiparity using the adjusted analyses described above. For 4 cohorts with the additional data, we conducted analyses that further adjusted for socioeconomic status, employment, and reproductive years. We also used adjusted logistic regression to investigate the association between parity as a continuous variable and the risk of all-cause dementia.

The effects of parity and geographic region, and their interaction, on the risk of all-cause dementia were investigated using a binary logistic regression. In this analysis, we grouped parity into nulliparity, 1 to 4 parities, and grand multiparity, and geographic region into Asia, Europe, and Latin America. We adjusted for age, educational level, hypertension, and DM.

We also conducted analyses within each geographic region that examined how both nulliparity and grand multiparity (vs 1 to 4 parities) were associated with (1) the risk of all-cause dementia, using binary logistic regressions, and (2) the risks of AD, VaD, and non-Alzheimer non-vascular dementia (NAVD), using multinomial logistic regressions. These analyses used data from the 9 cohorts with information on dementia subtype. We excluded individuals with mixed etiologies (2.5% of dementia) and adjusted for age, educational level, hypertension, DM, and cohort.

The KLOSCAD team harmonized and pooled the dataset and performed the analyses using the Statistical Package for Social Sciences, v20 (SPSS Inc., Chicago, IL).

## Results

The characteristics of the contributing cohorts are summarized in Table 1. At least 10% of women were grand multiparous in 5 cohorts and nulliparous in 4 cohorts. Grand multiparity was more frequent in the Latin American cohorts (28.6%) than in the Asian (17.8%) and European (7.8%) cohorts. The Asian cohorts had less frequent nulliparity (4.5%) than the European (14.3%) and Latin American (11.2%) cohorts (Table 2). The prevalence of dementia was also different between regions. The frequency of dementia was 10% or more in 3 cohorts, and higher in the Latin American cohorts compared to the Asian and European cohorts (*P* <  0.01). When the prevalence of dementia subtypes was compared separately, AD was less prevalent in the Asian cohorts compared to the European and Latin American cohorts while NAVD was more prevalent in the Latin American cohorts compared to the Asian and European cohorts (*P* <  0.01). Compared to women with 1 to 4 parities, grand multiparous women were older (72.3 ± 7.7 years vs 75.6 ± 7.6 years, *P* < 0.01), showed higher proportion of only primary or less education (44% vs 80%, *P* < 0.01), and had more hypertension (65% vs 71%, *P* < 0.01) and DM (17% vs 24%, *P* < 0.01).

**Table 2:** Comparison of the cohort characteristics between three geographical regions

Characteristics	Total (*N* = 14,792)	Asia (*N* = 5920)	Europe (*N* = 6171)	Latin America (*N* = 2701)	Statistics*		
					*F* or *χ*^2^	*P*	Post hoc*
Age, mean ± SD, years	73.1 ± 7.9	71.4 ± 7.8	74.2 ± 8.0	74.0 ± 7.0	217.79	< 0.001	a < b, a < c
Educational level, %					1049.72	< 0.001	–
≤ 6 years or primary school or under	48.8	46.6	39.4	75.0			
7 to 12 years or secondary school	38.8	39.0	48.4	16.1			
> 12 years or tertiary school or above	12.5	14.4	12.2	12.2			
Hypertension, %	66.5	55.7	71.2	79.2	569.22	< 0.001	a < b, a < c, b < c
Diabetes mellitus, %	17.3	16.6	12.7	29.5	375.205	< 0.001	a > b, a < c, b < c
Parity (continuous), mean ± SD	2.8 ± 2.2	3.0 ± 1.8	2.3 ± 1.6	3.8 ± 3.5	512.99	< 0.001	a > b, a < c, b < c
Parity (categorical), %					949.73	< 0.001	
0 (nulliparity)	9.8	4.5	14.3	11.2			
1 to 4	74.6	77.8	77.9	60.2			
≥ 5 (grand multiparity)	15.6	17.8	7.8	28.6			
Dementia prevalence, %	5.9	5.7	4.7	9.1	64.60	< 0.001	a < c, b < c
Dementia subtypes,^†^ %					54.14	< 0.001	–
AD	4.0	3.1	4.1	4.8			
VD	0.6	0.7	0.5	0.9			
NAVD	1.4	0.4	0.9	3.3			

*SD* standard deviation, *AD* Alzheimer’s disease, *VaD* vascular dementia, *NAVD* non-Alzheimer non-vascular dementia

*Continuous variables were compared using analysis of variance, and categorical variables were compared using the *χ*^2^ test. Post hoc comparisons were based on Scheffé’s theorem: a = Asia; b = Europe; c = Latin America

^†^Estimated using 9 cohorts providing data on dementia subtypes

The number of parities was associated with hypertension and DM (Table 3). Compared to women with 1 to 4 parities, grand multiparous women were more likely to have hypertension and DM (*P* < 0.01) and nulliparous women showed a higher frequency of hypertension and lower frequency of DM (*P* < 0.01).

**Table 3:** Comparison of the cohort characteristics between three parity groups

Characteristics	Number of parities			Statistics*		
	0	1 to 4	≥ 5	*F* or *χ*^2^	*P*	Post hoc*
Age, mean ± SD, years	74 ± 8.3	72.3 ± 7.7	75.6 ± 7.7	224.345	< 0.001	a > b, a < c, b < c
Educational level, %				1122.978	< 0.001	–
≤ 6 years or primary school or under	36.5	43.9	80.0			
7 to 12 years or secondary school	44.4	42.6	17.1			
> 12 years or tertiary school or above	19.1	13.6	2.9			
Hypertension, %	69.3	65.2	70.9	33.289	< 0.001	a > b, b < c
Diabetes mellitus, %	13.1	16.5	23.5	84.376	< 0.001	a < b, a < c, b < c,
Dementia prevalence, %	5.8	5.0	10.5	103.961	< 0.001	a < c, b < c,
Dementia subtypes,^†^ %				112.556	< 0.001	–
AD	4.6	3.1	7.1			
VD	0.6	0.5	1.1			
NAVD	2.2	1.0	2.4			

*Continuous variables were compared using analysis of variance, and categorical variables were compared using the *χ*^2^ test. Post hoc comparisons were based on Scheffé’s theorem: a = nulliparity; b = 1 to 4 parities; c = grand multiparity

^†^Estimated using 9 cohorts providing data on dementia subtypes

Table 4 shows the risk of all-cause dementia associated with parity determined with our initial analyses. In the unadjusted logistic regression model, the risk of all-cause dementia was not significantly different for nulliparous women and women with 2, 3, or 4 parities, but was significantly higher for grand multiparous women compared to primiparous women. In the logistic regression model adjusting for age, educational level, and cohort, grand multiparous women showed 1.47-fold higher risk of all-cause dementia than primiparous women (95% confidence interval [CI] = 1.11–1.94, *P* < 0.01). When we further adjusted for hypertension and DM, the association between grand multiparity and the risk for all-cause dementia remained significant (OR = 1.47, 95% CI = 1.10–1.94, *P* < 0.01). When we divided grand multiparous women into two groups, women with 5 to 9 parities and great grand multiparous women showed 1.44-fold (95% CI = 1.08–1.90, *P* = 0.01) and 1.86-fold (95% CI = 1.11–3.09, *P* = 0.02) higher risks of all-cause dementia, respectively. Further analyses with 1 to 4 parities as the reference group found the risk of all-cause dementia to be significantly higher in grand multiparous women (odds ratio [OR] = 1.33, 95% CI = 1.09–1.60, *P* < 0.01) but not significantly different in nulliparous women (OR = 0.83, 95% CI = 0.64–1.08, *P* = 0.16). The results were similar when additionally adjusting for economic status, employment, and reproductive years; grand multiparity was associated with an increased risk of all-cause dementia (OR = 1.46, 95% CI = 1.13–1.89, *P* < 0.01), but nulliparity was not (OR = 1.07, 95% CI = 0.70–1.62, *P* = 0.77). An analysis with parity as a continuous variable showed the risk of all-cause dementia to increase as the number of parities increased (OR = 1.06, 95% CI = 1.03–1.10, *P* < 0.01).

**Table 4:** The risk of all-cause dementia associated with parity

Parity	Number of subjects		Unadjusted model		Adjusted model I*		Adjusted model II^†^	
	Control	Dementia	OR (95% CI)	*P*	OR (95% CI)	*P*	OR (95% CI)	*P*
0 (nulliparity)	1368	84	1.25 (0.93–1.68)	0.014	0.91 (0.66–1.25)	0.560	0.92 (0.67–1.27)	0.613
1 (primiparity)	2096	103	1.00 [Reference]		1.00 [Reference]		1.00 [Reference]	
2	3923	181	0.94 (0.73–1.20)	0.618	1.12 (0.86–1.46)	0.397	1.13 (0.86–1.47)	0.375
3	2819	161	1.16 (0.90–1.50)	0.245	1.19 (0.91–1.57)	0.206	1.19 (0.91–1.57)	0.200
4	1652	103	1.27 (0.96–1.68)	0.096	1.06 (0.79–1.44)	0.690	1.07 (0.79–1.45)	0.664
≥ 5 (grand multiparity)	2061	241	2.38 (1.87–3.02)	< 0.001	1.47 (1.11–1.94)	0.007	1.47 (1.10–1.94)	0.007
5 to 9	1836	212	2.35 (1.84–3.00)	< 0.001	1.43 (1.08–1.90)	0.012	1.44 (1.08–1.90)	0.012
≥ 10	225	29	2.62 (1.70–4.05)	< 0.001	1.84 (1.11–3.06)	0.019	1.86 (1.11–3.09)	0.018

*OR* odds ratio, *CI* confidence intervals

*Binary logistic regression analyses adjusted for age, educational level, and cohort as covariates

^†^Binary logistic regression analyses adjusted for age, educational level, hypertension, diabetes mellitus, and cohort as covariates

In further analyses, the risk of all-cause dementia was significantly associated with parity (*P* < 0.01) and geographic region (*P* < 0.01), as well as their interaction (*P* < 0.01). As seen in Table 5, compared to women with 1 to 4 parities, grand multiparous women showed about 3-fold and 1.5-fold higher risk of all-cause dementia in the European (*P* < 0.01) and Latin American (*P* = 0.03) cohorts. Grand multiparous women also showed a modestly higher risk of all-cause dementia in the Asian cohorts, but this was not statistically significant (*P* = 0.24). Nulliparous women showed about 2-fold higher risk of all-cause dementia than women with 1 to 4 parities in the Asian cohorts (*P* < 0.01), but associations with nulliparity were not statistically significant in the European (*P* = 0.66) and Latin American (*P* = 0.26) cohorts.

**Table 5:** The risks of nulliparity and grand multiparity for dementia according to subtypes and geographical regions

	Numbers of subjects					All-cause dementia		AD		VaD		NAVD	
	Control	Dementia	AD	VaD	NAVD	OR (95 CI)*	*P*	OR (95 CI)*	*P*	OR (95 CI)*	*P*	OR (95 CI)*	*P*
Nulliparity													
Asia	239/5584	27/336	17/244	1/27	7/54	2.15 (1.33–3.47)	0.002	1.91 (1.07–3.39)	0.028	1.03 (0.13–7.89)	0.977	3.47 (1.44–8.35)	0.005
Europe	117/3098	25/125	12/88	1/22	1/12	0.70 (0.38–1.30)	0.261	0.90 (0.46–1.76)	0.761	0.28 (0.04–2.13)	0.218	0.53 (0.06–4.32)	0.552
Latin America	2456/270	32/245	15/129	4/24	13/89	0.91 (0.59–1.40)	0.661	0.72 (0.40–1.30)	0.277	1.39 (0.42–4.57)	0.585	1.11 (0.58–2.14)	0.746
Grand multiparity													
Asia	919/5584	132/336	102/244	10/27	1/54	1.25 (0.94–1.67)	0.122	1.29 (0.93–1.79)	0.127	1.37 (0.55–3.42)	0.498	1.22 (0.61–2.43)	0.581
Europe	117/3098	39/125	6/88	3/22	1/12	2.99 (1.38–6.47)	0.005	2.42 (0.94–6.25)	0.068	6.86 (1.81–26.08)	0.005	3.36 (0.37–30.80)	0.283
Latin America	702/2456	70/245	30/129	9/24	28/89	1.49 (1.04–2.12)	0.028	1.25 (0.77–2.03)	0.366	1.41 (0.53–3.79)	0.495	1.80 (1.05–3.10)	0.034

*AD* Alzheimer’s disease, *VaD* vascular dementia, *NAVD* non-Alzheimer non-vascular dementia, *OR* odds ratio, *CI* confidence intervals

*Binary or multinomial logistic regression analyses that employed the women with 1 to 4 parities as the referent group and adjusted age, educational level, hypertension, diabetes mellitus, and cohort as covariates using the pooled dataset of 9 cohort studies that provided the type of dementia (Cuban Health and Alzheimer Study, Etude SanteÂ Psychologique et Traitement, Gothenburg H70 Birth cohort Studies, Hellenic Longitudinal Investigation of Aging and Diet, Korean Longitudinal Study on Cognitive Aging and Dementia, Kurihara Study, Leipzig Longitudinal Study of the Aged, Shanghai Aging Study, Sao Paulo Ageing & Health Study)

The results of analyses examining how grand multiparity and nulliparity were associated with the risks of AD, VaD, and NAVD separately in each geographic region are also shown in Table 5. Compared to women with 1 to 4 parities, grand multiparity was associated with a more than 6-fold higher risk of VaD in the European cohorts (*P* < 0.01) and about a 1.8-fold higher risk of NAVD in the Latin American cohorts (*P* = 0.03). Nulliparity was associated with about a 1.9-fold higher risk of AD (*P* = 0.03) and about a 3.5-fold higher risk of NAVD (*P* < 0.01) in the Asian cohorts. However, the associations between nulliparity and the risk of AD, VaD, and NAVD were not statistically significant in other geographic regions.

## Discussion

This study included nearly 15,000 community-dwelling elderly women from 11 population-based prospective cohort studies from 11 countries over 3 continents and found that grand multiparity and nulliparity were associated with an increased risk of dementia. However, these associations were not uniform across different geographic regions or dementia subtypes. Grand multiparity was associated with an increased risk of VaD in European cohorts and an increased risk of NAVD in Latin American cohorts. Conversely, nulliparity was associated with an increased risk of both AD and NAVD in Asian cohorts.

The association between grand multiparity and the risk of all-cause dementia has not been previously investigated. Gilsanz et al., using a claims database that included 14,595 women (Caucasian 68%, African American 16%, Asian 6%, and Hispanic 5%), reported that women with 3 or more childbirths showed modestly lower risk of all-cause dementia (hazard ratio = 0.88, 95% CI = 0.81–0.95) than primiparous women [13]. However, they did not specifically analyze the association between grand multiparity and the risk of all-cause dementia, and their dementia diagnoses were derived from the billing codes of electronic medical records, which may lack diagnostic accuracy [34]. Their results thus cannot be directly compared against our finding that grand multiparity increased the risk of dementia. It has been previously reported that grand multiparous women are more likely to have risk factors for dementia that include being less educated, unemployed, and economically disadvantaged [6], experiencing more stress during childbearing, and higher rates of medical conditions such as coronary heart disease, stroke, DM, and metabolic syndrome [7–9]. This is consistent with the data we had showing less education and higher rates of hypertension and DM in grand multiparous women. Repetitive pregnancies and childbirths may also have direct cumulative harmful effects on the brain. For example, serum estradiol increases more than 10-fold and insulin sensitivity drops to 50% in the third trimester, with both effects potentially neurotoxic [35, 36]. Gray matter volumes in multiple areas including the hippocampus were reduced and persisted for 2 years after birth [37]. Density and numbers of microglia which is known to contribute to neurogenesis and spinogenesis were reduced during peripartum period, and its proliferation was reduced in the postpartum hippocampus [38]. In addition, serum estrogen was reduced after pregnancy in both premenopausal and postmenopausal periods [39, 40].

The number of people with dementia is expected to more than double in Africa, Asia, and Latin America, and the global proportion of individuals with dementia living in these regions will have increased to about 70% by 2050 [41]. Women in these regions have typically had much higher fertility rates than women in Europe and North America [42]. Despite the global fertility rate having fallen by half in the last 60 years or so (from 5 to 2.5 births per woman), it remains at around five in most African countries [41]. While this suggests that grand multiparity may be a risk factor for dementia of particular importance to Africa, further research is needed given our lack of African cohorts and findings of geographic differences.

Although grand multiparity was associated with an increased risk of dementia both in European and Latin American cohorts, the association was not significant in Asian cohorts. There were also differences associated with dementia subtype. Grand multiparity was associated with a more than 6-fold higher risk of VaD in European cohorts, and about a 2-fold risk of NAVD in Latin American cohorts. Different associations between grand multiparity and dementia risk by geographic region and dementia subtype might be due to different ethno-racial susceptibilities of the hormonal and vascular system to the effects of pregnancy or childbirth. For example, it has been previously reported that the number of parities influenced gestational period estradiol levels in Caucasian women, but not in Asian women [43], and that the risks of gestational hypertension and preeclampsia were higher in Caucasian women than in Asian/Pacific Islanders and Hispanic women [44]. Insulin resistance, which was more prevalent in postmenopausal women with 4 or more children compared to those with 0–3 parities [45], may influence the risk of dementia in European regions where DM was less prevalent compared to the Asian and Latin American regions. Although we could not adjust the effect of apolipoprotein E genotype in the present study, different distribution of apolipoprotein E genotype and psychosocial stresses associated with childbearing between regions may possibly contribute to the different association between grand multiparity and the risk of dementia between regions.

In contrast to grand multiparity being associated with an increased risk of dementia in both the European and Latin American cohorts, only in Asian cohorts was nulliparity associated with a higher risk of dementia, particularly non-vascular dementia. In the current study, the proportion of VaD in all-cause dementia was higher in the European cohorts (18%) than in Asian (8.3%) and Latin American (9.9%) cohorts. This inter-regional difference in the association between nulliparity and the risk of dementia may be, at least in part, attributable to an inter-regional difference in the causes of nulliparity. In the current study, the proportion of nulliparous women in Asian cohorts (4.5%) was much lower than that in European (14.3%) and Latin American (11.2%) cohorts, and nonmarriage and voluntary childlessness in nulliparous women are less common in Asian countries compared to the Western countries [46]. Therefore, nulliparous women in Asia are more likely to have involuntary factors as causes of nulliparity. The majority of involuntary causes of nulliparity are infertility and recurrent miscarriage. The most common cause of infertility is ovulatory dysfunction caused by premature ovarian failure, ovarian hyperandrogenism, hypothalamic dysfunction, pituitary disease, and thyroid disease [47], and these conditions are reportedly associated with the risk of cognitive impairment or AD [48]. Furthermore, women experiencing recurrent miscarriage are more likely to have the apolipoprotein E ε4 allele, which conveys an increased risk of AD [49].

The current study has limitations. First, we could not adjust for some well-known associated factors for dementia such as apolipoprotein E genotype, diet, reproductive experiences such as breast feeding, and gynecological surgery that could be associated with parity. However, in our previous work, the association between parity and AD remained significant even after adjusting for breast feeding, history of hormonal replacement therapy, hysterectomy, and oophorectomy [10]. Second, the data harmonization procedure might have confounded our observations. For example, parity data was based on the number of childbirths in 6 cohorts and the number of children in 5 cohorts, and adoption or death of children might have distorted parity information. However, this would likely apply to only a very small number of cases, given that for our KS, only 0.3% of women had different numbers for childbirths and children. Third, information on parity was obtained retrospectively. However, childbirths are life events not easily forgotten and likely to be minimally prone to recall bias. Fourth, regional difference in the diagnostic rates of dementia and its subtypes might have influenced the differential associations of parity with dementia subtypes according to geographical regions because the prevalence of dementia subtypes was considerably different between geographical regions. Fifth, women with middle- and late-stage dementia might be less likely to be included in the present study because all participants were community-dwelling.

## Conclusions

Grand multiparity and nulliparity are associated with the risk of dementia in women, but these associations were not uniform across geographic regions and dementia subtypes. The findings of this study help to understand the development of dementia in women, and have important implications for world regions where the birth rate remains high.

## Data Availability

The datasets used and/or analyzed during the current study are available from the corresponding author on reasonable request.

## Abbreviations

AD: Alzheimer disease
CHAS: Cuban Health and Alzheimer Study
CI: Confidence interval
COSMIC: Cohort Studies of Memory in an International Consortium
DM: Diabetes mellitus
ESPRIT: Etude SanteÂ Psychologique et Traitement
H70: Gothenburg H70 Birth Cohort Studies
HELIAD: Hellenic Longitudinal Investigation of Aging and Diet
KLOSCAD: Korean Longitudinal Study on Cognitive Aging and Dementia
KS: Kurihara Study
LEILA75+: Leipzig Longitudinal Study of the Aged
MAAS: Maastricht Ageing Study
NAVD: Non-Alzheimer non-vascular dementia
OR: Odds ratio
SAS: Shanghai Aging Study
SPAH: Sao Paulo Ageing & Health Study
VaD: Vascular dementia
ZARADEMP: Zaragoza Dementia Depression Project
